# Electrical and Electrorheological Properties of Alumina/Natural Rubber (STR XL) Composites

**DOI:** 10.3390/ma3010656

**Published:** 2010-01-22

**Authors:** Nuchnapa Tangboriboon, Nuttapot Uttanawanit, Mean Longtong, Piraya Wongpinthong, Anuvat Sirivat, Ruksapong Kunanuruksapong

**Affiliations:** 1 Department of Materials Engineering, Faculty of Engineering, Kasetsart University, Bangkok 10900, Thailand; E-Mails: fengnnpt@ku.ac.th (N.T.); nuttapoth@hotmail.com (N.U.); uv3w@hotmail.com (M.L.); narinninja@hotmail.com (P.W.); 2 The Petroleum and Petrochemical College, Chulalongkorn University, Bangkok 10330, Thailand; E-Mail: amd64best@hotmail.com

**Keywords:** natural rubber, dielectric elastomer, alumina

## Abstract

The electrorheological properties (ER) of natural rubber (XL)/alumina (Al_2_O_3_) composites were investigated in oscillatory shear mode under DC electrical field strengths between 0 to 2 kV/mm. SEM micrographs indicate a mean particle size of 9.873 ± 0.034 µm and particles that are moderately dispersed in the matrix. The XRD patterns indicate Al_2_O_3_ is of the β-phase polytype which possesses high ionic conductivity. The storage modulus (G′) of the composites, or the rigidity, increases by nearly two orders of magnitude, with variations in particle volume fraction and electrical field strength. The increase in the storage modulus is caused the ionic polarization of the alumina particles and the induced dipole moments set up in the natural rubber matrix.

## 1. Introduction 

Combining a dielectric ceramic and a polymer host to form a flexible dielectric composite is interesting in view of the greater flexibility in tailoring these materials towards particular mechanical, electrical, and thermal properties, and/or a coupling between these properties [1,2]. Carpi *et al.* reported the actuation performances of elastomeric dielectric materials used as electromechanical transducers [3]. López-Manchado *et al.* studied the influence of inorganic nanoparticles on elastomer crosslinking mechanisms. The role of inorganic particles has been evaluated using the tube model theory [4]. Molecular network parameters derived from this model point out a different filler/elastomer reinforcement mechanism as a function of filler. Even a small amount of inorganic nanoparticles is sufficient to interact with the entire matrix. Furthermore, these inorganic nanoparticles mainly exhibit physical adsorptions with the elastomer due to its inorganic nature [4]. 

The backbone of natural rubber (NR), polyisoprene, is derived from the polyacetylene backbone through the saturation of every other double bond. Polyisoprene is a potential candidate for materials used in various devices: solar cells, LEDs, and FETs. Natural rubbers combine high tensile strength, flexibility, and tear strength with outstanding resistance to fatigue [5,6]. External stimuli, including electrical fields, temperature, and stress may cause the methyl substitute attached to the unsaturated sites in polyisoprene to induce the electron-releasing tendency of the backbone. The pristine polyisoprene chains generally have a random coil conformation in various organic solvents over a range of concentrations. However, natural rubber (NR), unlike many other polymers, is highly susceptible to many forms of degradation: heat, humidity, light, ozone, and radiation, due to the presence of double bonds in the main chain. 

Alumina is a linear dielectric material with ionic polarization with potential uses as electrolytic capacitors, optical and photoluminescent materials [7]. The most common form of crystalline alumina is corundum, which has a rhombohedral Bravais lattice with a space group R-3c (Number 167 in the International Tables). Alumina also exists in other phases, namely η, γ, θ, and δ theta alumina. All phases have a structure with a spinel-like Al-O network similar to that found in the β-alumina polytypes. Sutorik *et al*. reported that β- and β"-alumina are high temperature solid electrolytes that exhibit high ionic conductivity. These materials have been used in energy storage, alkali-metal thermal-to-electric conversion (AMTEC) cells, and gas sensors [8]. Many of the challenges in working with β- and β"-alumina result from the desire to optimize the formation of β"-alumina (which has the higher ionic conductivity) over that of β-alumina. The structures of β- and β"-alumina are closely related polytypes; both have the same general stacking pattern of Al-O spinel-like blocks separated by Na-O planes [7,9]. β-alumina has the interlayer Na-O lying on a crystallographic mirror plane and two Al-O blocks per unit cell. β"-alumina, rather, has a three-fold screw axis perpendicular to the planes and consequently possesses three Al-O blocks in its unit cell. In general, the properties of alumina (Al_2_O_3_) are a low thermal-expansion, a good thermal-shock fracture resistance, a low density, high creep resistance, good chemical and thermal stability, a high melting point (1,828 ± 10 °C), and excellent toughness and strength. Vinod *et al.* and Jakubowickz reported that alumina can also reduce heat, humidity, light, ozone and gamma radiation, flame resistance, and the crack growth in natural rubber [10,11]. Alumina (Al_2_O_3_) is a potential candidate as the dispersed phase in the natural rubber matrix [12,13]. Factors such as size distribution, shape, volume fraction, permittivity, and conductivity of the particles and the host solvent, have been well known to affect the electrical and rheological properties of the polyisoprene based blends or composites [14,15,16,17,18,19,20,21]. Composite potential applications are the dissipation of an electrostatic charge; the electrostriction, the ion insertion, and molecular conformational changes towards the development of friction/anti-friction materials, and the responses from external stimuli such as changes in pH, temperature, time, light, ionic concentration or electrical field [14,15,16,17,18,19,20,21]. Recently, several designs based on electrorheological (ER) materials have led to the developments of a broad range of devices: brakes, dampers, clutches, adaptive structures, actuators, artificial muscles, prostheses, toys, robotics, and biomimetic devices [5,14,22,23,24,25].

The aim of this work was to study the electrical and electrorheological properties of composites based on alumina particles (the dispersed phase) embedded in a dried natural rubber type XL (matrix or the continuous phase). In addition, detailed characterizations by SEM, FTIR, and XRF of composites, the matrix, and the dispersed phase shall be reported. 

**Figure 1:**
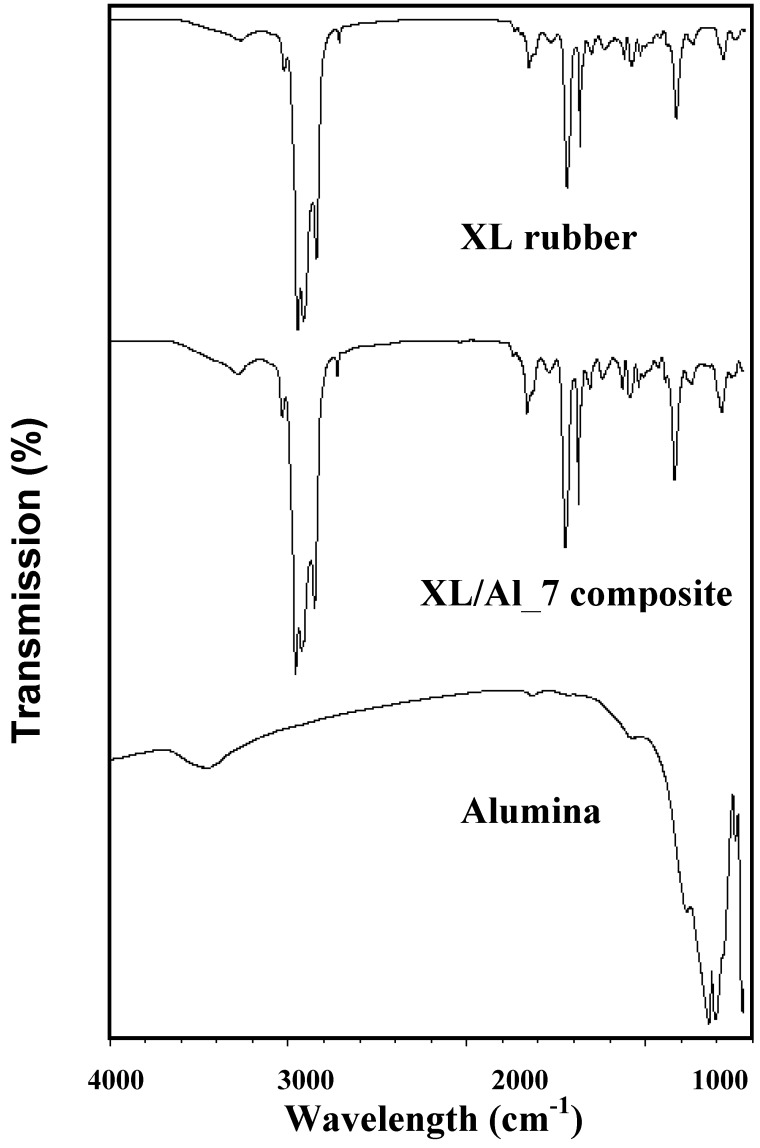
FTIR spectra: natural rubber (XL), alumina (Al_2_O_3_), and XL/Al_2_O_3_ composites.

## 2. Results and Discussion 

### 2.1. Characterization of Natural Rubber (XL)/Alumina (Al_2_O_3_)

The mean diameter of alumina particles is 9.873 ± 0.034 µm, as determined by the particle size analyzer, and consistent with that found by SEM. The chemical composition of alumina powder, as measured by the X-ray fluorescence is 99.74% (that is, 0.26% impurity content). The FTIR spectra of alumina (Al_2_O_3_), natural rubber (XL) and the XL/Al_2_O_3_ composites are shown in Figure 1; the peaks are identified and tabulated in Table 1. 

**Table 1:** FTIR spectra of natural rubber (XL), alumina (Al), and XL/Al_2_O_3_ composites.

Wave Number (cm^-1^)	Functional Groups
3281, 3467 (CAS7732-18-5); (CAS78-79-5)	OH stretching of Al_2_O_3_ added natural rubber (XL)
2961, 3036 (CAS78-79-5)	-CH and =CH stretching vibration of poly-acetylene backbone of isoprene
2855, 2928 (CAS78-79-5)	-CH_2_ stretching vibration of C-CH_2_CH_3_
1663,1710 (CAS78-79-4)	>C=C stretching for carbon-carbon double bond
1448 (CAS78-79-5); (CAS110-54-3); (CAS73513-42-5)	- CH_3_ asymmetric deformation
1376 (CAS78-79-5)	- CH_3_ deformation of O-CH_2_CH_3_
1242, 1288 (CAS110-54-3)	Asymmetric C-O-C stretching vibration
1128	R-CO-R symmetric stretching
740, 762,1038,1084 (CAS110-54-3)	Skeletal vibration of hexane and =CH bending
572, 639	Al-O-C stretching

**Figure 2:**
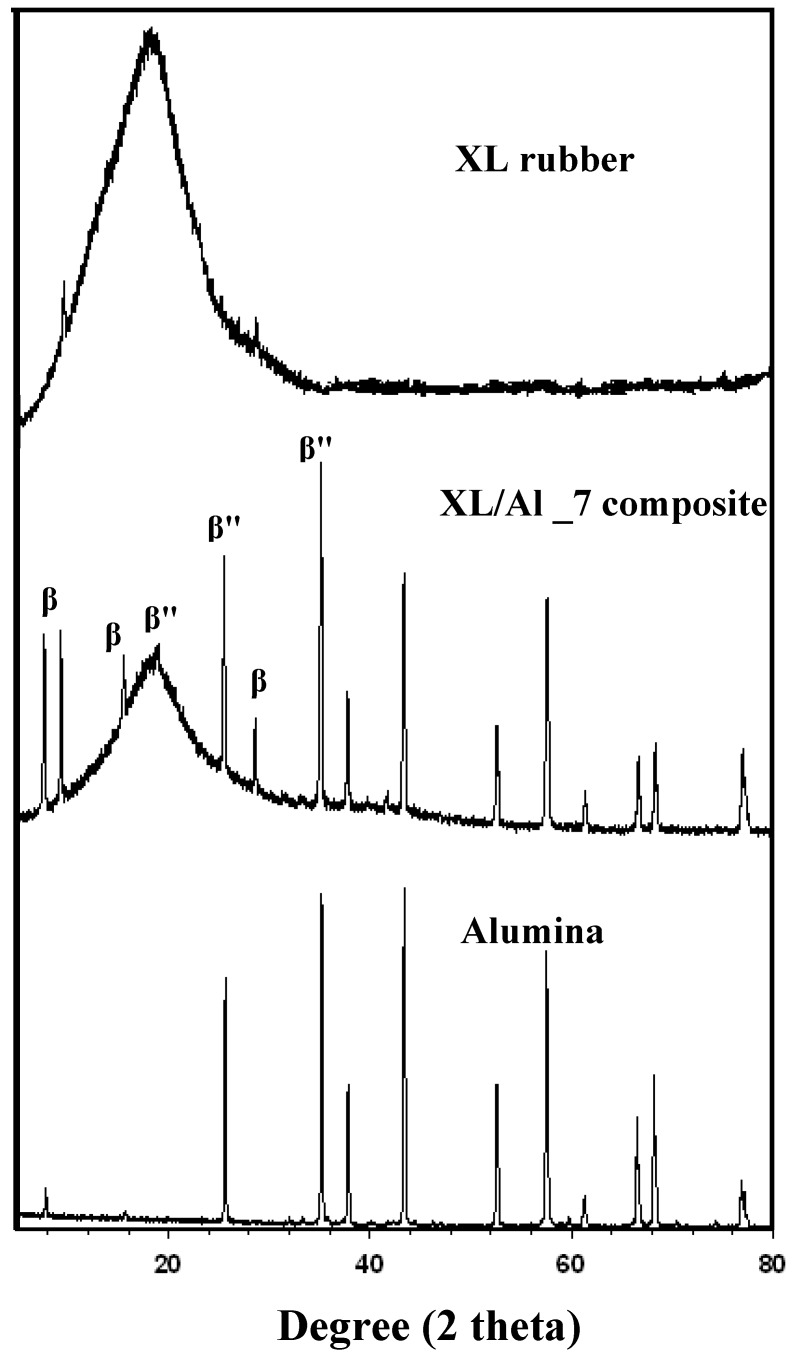
XRD peak patterns of natural rubber (XL), alumina (Al_2_O_3_), and XL/Al_2_O_3_ composites.

The characteristic peaks of XL/Al_2_O_3_ are at 3,600–3,200 cm^-1^ ν(O-H), 3,000–2,900 cm^-1^ ν(C-H) and (=CH), 2,900–2,800 cm^-1^ ν(CH_2_), 1,800–1,600 cm^-1^ ν(C=C), 1,500–1,300 cm^-1^ (CH_3_ asymmetric deformation), 1,300–1,100 cm^-1^ ν(C-O-C), 1,100–740 cm^-1^ (=CH bending), and 640–580 cm^-1^ ν(Al-O). Our FTIR result is consistent with those reported by Ismail *et al*. [24] and Kansal *et al.* [28]. The XRD patterns of XL, Al_2_O_3_, and XL/Al_2_O_3_, are shown in Figure 2. 

**Figure 3:**
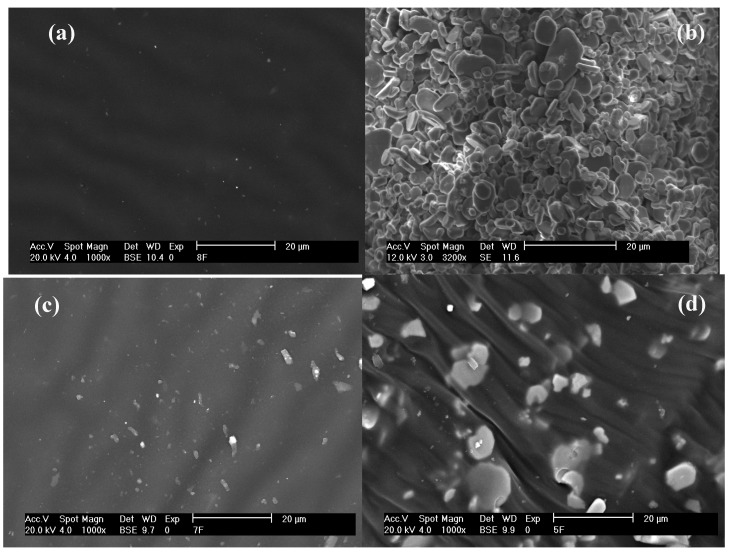
SEM micrographs of samples: (a) Natural rubber (XL), (b) Alumina powder (Al_2_O_3_), (c) XL/Al_6, and (d) XL/Al_7.

The XRD patterns of alumina powder and XL/Al_2_O_3_ resemble those recorded at the International Center for Diffraction Data (JCPDS numbers 00-042-1458 with the corundum phase form and 01-070-0384 with the hexagonal phase form). This observation indicates a partial phase transformation from the corundum phase (rhombohedral) to β- and β"-alumina forms (hexagonal). The structures of β- and β"-alumina are the closely related polytypes; both have the same general stacking pattern of Al-O spinel-like blocks separated by Na-O planes [7]. The β- and β"-alumina forms exhibit high ionic conductivity; impurity ions (K^+^, Ca^2+^, and Na^+^) as traced by XRF can migrate through a surplus of cationic sites in the crystal lattice. The microstructures of pure natural rubber (XL), XL/Al_6, and XL/Al_7, and alumina particles are shown in SEM micrographs of Figure 3. The particle size distribution and the shapes of the particles have a significant effect on the particle packing and pore structure, on the deformation behavior, and on the electrical and rheological properties. The calculated and experimental density values of XL/Al_2_O_3_ composites are: 0.9200 and 0.9428 g/cm^3^ (XL/Al_0); 0.9216 and 0.9449 g/cm^3^ (XL/Al_1); 0.9274 and 0.9527 g/cm^3^ (XL/Al_2); 0.9333 and 0.9607 g/cm^3^ (XL/Al_3); 0.9383 and 0.9674 g/cm^3^ (XL/Al_4); 0.9424 and 0.9730 g/cm^3^ (XL/Al_5); 0.9460 and 0.9780 g/cm^3^ (XL/Al_6); and 0.9489 and 0.9818 g/cm^3^ (XL/Al_7). 

### 2.2. Electrical Properties of Natural Rubber (XL)/Alumina (Al_2_O_3_)

**Figure 4:**
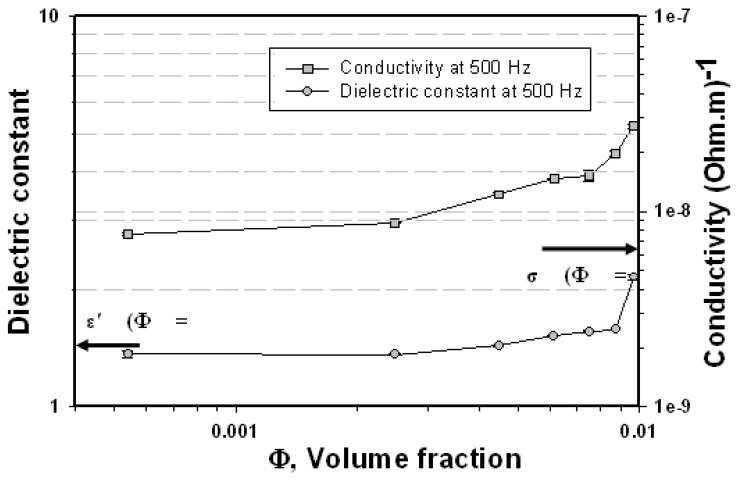
Electrical properties of Al_2_O_3_ and XL/Al_2_O_3_ composites *vs.* volume fraction.

The electrical properties of the non-conducting natural rubber (XL), the insulating material (Al_2_O_3_), and XL/Al_2_O_3_ composites were measured by the impedance analyzer; the data are tabulated in Table 2 and shown in Figure 4. With increasing particulate volume fraction, the electrical conductivity and the dielectric constant increase gradually. The dielectric constant values are 1.542, 11.811, and 2.123 for XL, Al_2_O_3_, and XL/Al_7, respectively. The electrical conductivity values of XL, Al_2_O_3_, and XL/Al_7 are 6.517 × 10^-9^, 1.502 × 10^-7^, and 2.683 × 10^-8^ (Ω.m)^-1^, respectively. The electrical conductivity of XL/Al_7 composite is approximately two orders of magnitude higher than that of the natural rubber XL. The polyisoprene can be swollen by the hexane, and the alumina is intercalated in the natural rubber XL matrix. The alumina has a partial phase transformation from rhombohedral to the hexagonal β- and β"- phase formations as shown by the XRD pattern. The β- and β"- alumina phases have high ionic conductivity [7]. The effect of a methyl group on the double bond of natural rubber XL is to stabilize the cationic structure. The ionic polarization mechanism of XL/Al_2_O_3_ acts as Al-O spinel-like blocks in Na-O planes under an applied AC electrical field.

**Table 2:** Electrical properties of XL/Al_2_O_3_ measured at room temperature and at 500 Hz.

Code	Mass of XL (g)	Mass of Al_2_O_3_ (g)	Volume fraction of Al_2_O_3_ (Φ)	Dielectric constant ε′ at 500 Hz	Dielectric loss factor ε″ at 500 Hz	Conductivity (Ω.m)^-1^, σ at 500 Hz
Al_2_O_3_	0.0000	1.0000	1.00000	11.811	7.66E-03	1.502E-07
XL/Al_0	3.0000	0.0000	0.00000	1.542	6.84E-04	6.517E-09
XL/Al_1	3.0000	0.0069	0.00054	1.343	2.15E-04	7.685E-09
XL/Al_2	3.0000	0.0316	0.00248	1.357	1.44E-03	8.688E-09
XL/Al_3	3.0000	0.0572	0.00448	1.436	1.36E-03	1.235E-08
XL/Al_4	3.0000	0.0784	0.00613	1.518	1.52E-03	1.471E-08
XL/Al_5	3.0000	0.0961	0.00750	1.550	1.58E-03	1.594E-08
XL/Al_6	3.0000	0.1120	0.00873	1.575	1.96E-04	1.937E-08
XL/Al_7	3.0000	0.1243	0.00968	2.123	1.65E-04	2.683E-08

Measured at 27 °C; Density XL is 0.92 g/cm^3^; Density Al_2_O_3_ powder is 3.90 g/cm^3^; XL rubber has extra light color.

### 2.3. Electrorheological Properties: Time Dependence, Effects of Particle Concentration and Electric Field Strength

**Figure 5:**
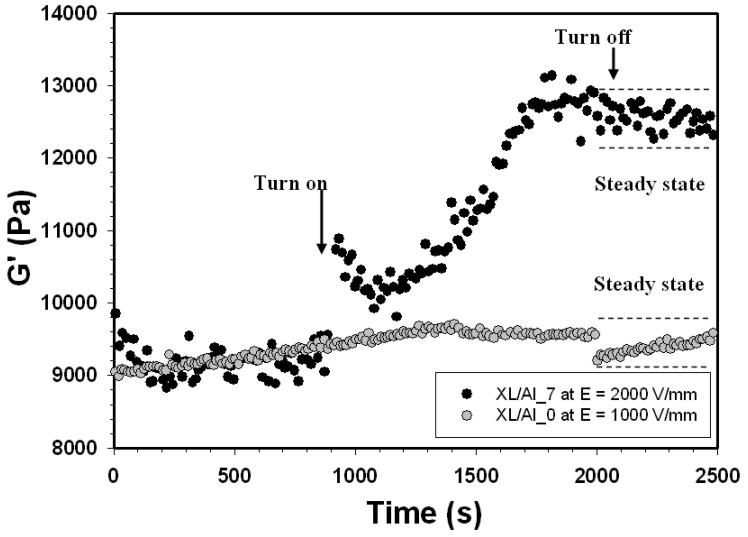
Temporal response of the storage modulus (G′) of XL/Al_0 and XL/Al_7 at electrical field strengths of 1 and 2 kV/mm, frequency 1.0 rad/s, strain 0.1%, and at 27 °C.

Figure 5 shows the temporal characteristics of pure natural rubber XL (XL/Al_0) and XL/Al_7 from time sweep tests in which an electrical field was alternately turned on and off. The temporal characteristics of the samples were recorded in the linear viscoelastic regime at electrical field strengths of 1 and 2 kV/mm, and a frequency of 1 rad/s. After an initial period with the electrical field on and off, at an electrical field strength of 1 kV/mm, the storage modulus G' (ω = 1 rad/s) of XL/Al_0, increases and subsequently reaches a steady-state value. While the storage modulus of XL/Al_7 initially increases, and then decreases slowly as the electrical field is turned off; it does not recover its original value. This behavior indicates that there are some irreversible interactions, perhaps due to the ionic bonding between small alumina particles within the matrix.

**Figure 6:**
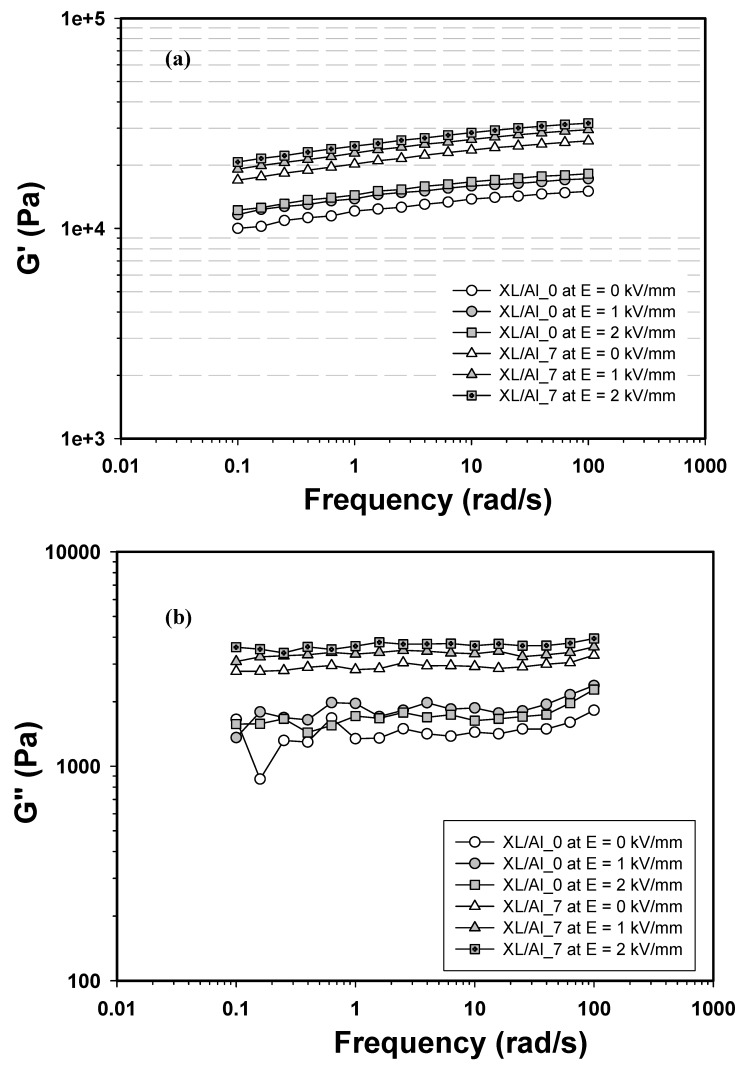
Moduli of XL/Al_0 and XL/Al_7 *vs.* frequency at various electrical field strengths: 0, 1, and 2 kV/mm; and at 27°C: a) Storage modulus, G′(ω); b) Loss modulus, G″(ω).

Figure 6 (a) and (b) show the storage modulus (G′) and the loss modulus (G″) *vs.* frequency of the XL/Al_0 and XL/Al_7 composites at the electrical field strengths of 0, 1, and 2 kV/mm. In the absence of an electrical field, G′ is greater than G″ at all frequencies for both XL/Al_0 and XL/Al_7 systems, indicating that these samples possess the solid-like behavior. A composite system with a volume fraction of 0.00968 (XL/Al_7) exhibits the highest electrorheological responses, the storage modulus G′(ω) and the loss modulus G″(ω), under the stimulation of an external electrical field. For the XL/Al_0 system, the G′ (ω = 1 rad/s) increases from ~1.16 × 10^4^ Pa to 1.25 × 10^4^ Pa as the electrical field is varied from 0 to 2 kV/mm; ΔG' = 0.09 x 10^4^ Pa. For the XL/Al_7 system, the G′ (ω = 1 rad/s) increases from ~2.00 × 10^4^ Pa to 2.44 × 10^4^ Pa as the electrical field is varied from 0 to 2 kV/mm; ΔG′ = 0.44 × 10^4^ Pa. The storage modulus of the composite without alumina (XL/Al_0) increases significantly upon electrical fields from 0 to 2 kV/mm because natural rubber (XL) is an elastic elastomer. The XL elastic behavior is caused by bond distortions. In its relaxed state rubber consists of long, coiled-up polymer chains that are interlinked and rotate freely about its neighbor. As an electrical field is applied, bond lengths deviate from the minimum energy equilibrium and the strain energy is stored electrostatically. 

**Figure 7:**
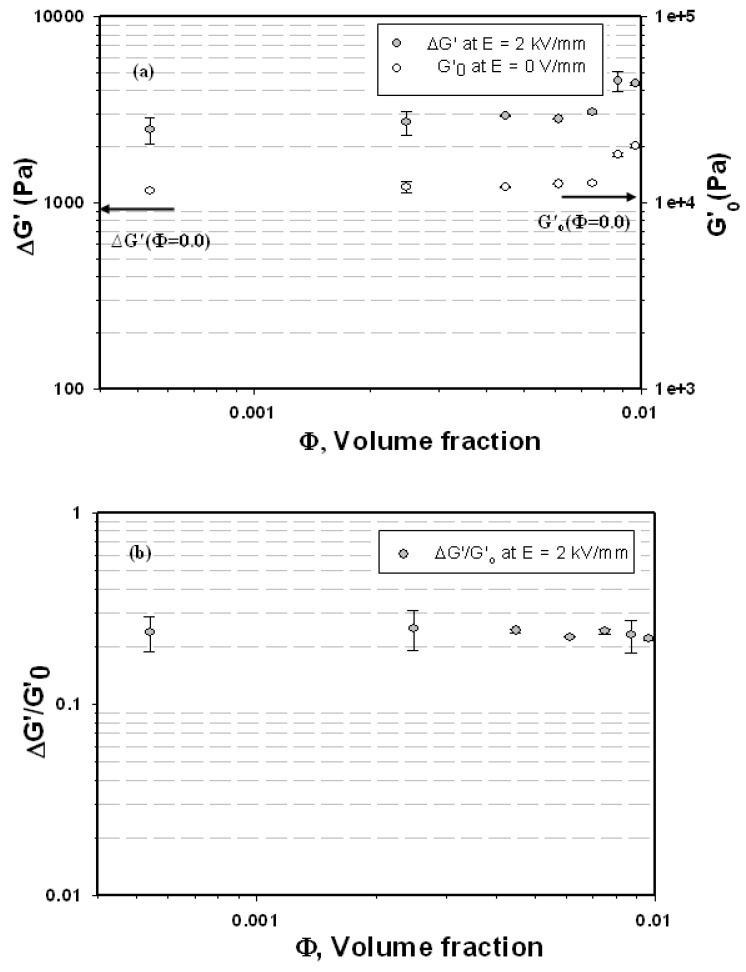
(a) Storage modulus response, ΔG′ (ω=1 rad/s, 2 kV/mm) and G′_ο_(ω=1 rad/s), of the XL/Al_7 composites as functions of particle volume fraction at 27 °C. (b) ΔG′(ω)/G′_ο_(ω) of the XL/ Al_2_O_3_ composite as a function of particle volume fraction at an electrical field strength of 2 kV/mm, and at a temperature of 27 °C.

Figure 7 (a) shows the storage modulus response, ΔG′(ω =1 rad/s), *vs.* particle volume fraction at the electrical field strengths of 2 kV/mm. In this figure, we also show the storage modulus, G′_ο_(ω =1 rad/s), without an electrical field versus particle volume fraction. Both ΔG′and G′_ο_ appear to increase monotonically with increasing particle volume fraction. For the storage modulus G′_ο_(ω = 1 rad/s) without an electrical field, the storage modulus increases from 11,590 Pa to 20,011 Pa as the volume fraction is varied from 0.00000 to 0.00968, as also tabulated in Table 3. Figure 7(b) shows the storage modulus sensitivity, ∆G′/G′_ο_, *vs.* particle volume fraction**.** The storage modulus sensitivity values at an electrical field strength of 2 kV/mm are 7.8854% (XL/Al_0), 23.6483% (XL/Al_1), 24.6102% (XL/Al_2), 24.1534% (XL/Al_3), 22.3179% (XL/Al_4), 23.9230% (XL/Al_5), 22.8915% (XL/Al_6), and 21.8755% (XL/Al_7). The storage modulus sensitivity of our composites is thus nearly independent of particle volume fractions; but it is higher than that of pure natural rubber (XL/Al_0). 

**Figure 8:**
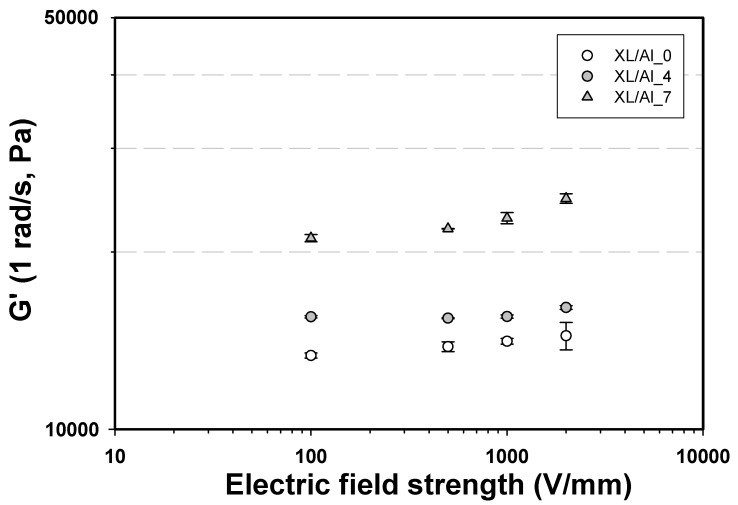
Storage modulus, G′(ω=1 rad/s), of XL/Al_0, XL/Al_4, and XL/Al_7 *vs*. electrical field strength, at a frequency of 1 rad/s, and at 27 °C.

The storage moduli G′(ω =1 rad/s) *vs.* electrical field of the three composites (XL/Al_0, XL/Al_4, and XL/Al_7) at various electrical field strengths are shown in Figure 8. G′(ω =1 rad/s) increases monotonically with electrical field within the range of 0.005–2 kV/mm. The storage modulus values, G′(ω =1 rad/s), of these systems at an electrical field strength of 2 kV/mm are 14411, 16184, and 24656 Pa for XL/Al_0, XL/Al_4, and XL/Al_7, respectively. In the absence of an electrical field, the alumina particles are randomly dispersed within the natural rubber XL matrix and there is no particle interaction. As an electrical field is applied, both alumina particles and XL particles become ionically polarized and induced dipole moments are generated, leading to intermolecular interactions. These intermolecular interactions induce the loss of chain free movements and higher chain rigidity, as indicated by higher G′(ω) values. The electrical field evidently enhances the elastic modulus of our dielectrical ceramic-polymer composite materials by nearly a factor of two.

**Table 3:** Rheological properties of composite materials of natural rubber (XL) and alumina (Al_2_O_3_) measured at 1 rad/s and at 27 °C.

Code	V/V of Al_2_O_3_	G′_ο_ (Pa)	G′_2 kV/mm_ (Pa)	G″_ο_ (Pa)	G″_2 kV/mm_ (Pa)	ΔG′_2 kV/mm_ (Pa)	ΔG′_2 kV/mm_/G′_ο_
XL/Al_0	0.00000	11,589	12,503	1,344	1,476	913	0.0788
XL/Al_1	0.00054	11,677	14,439	1,458	1,565	2,761	0.2364
XL/Al_2	0.00248	12,704	15,831	1,537	1,609	3,126	0.2461
XL/Al_3	0.00448	12,269	15,233	1,559	1,689	2,963	0.2415
XL/Al_4	0.00613	12,634	15,454	1,609	2,263	2,819	0.2231
XL/Al_5	0.00750	12,736	15,783	2,076	2,339	3,046	0.239
XL/Al_6	0.00873	17,941	22,048	2,080	2,640	4,107	0.2289
XL/Al_7	0.00968	20,010	24,387	2,826	3,638	4,377	0.2187

All properties were measured at the frequency 1 rad/s, the strain of 0.1%, and the temperature of 27 °C. G′_ο_ and G″_o_ are the storage and the loss moduli without an electrical field; G′_2kV/mm_ and G″_2kV/mm_ are the storage and the loss moduli at 2kV/mm; ΔG′_2 kV/mm_ is the storage modulus response defined as G′_2kV/mm_ - G′_ο_; ΔG′_2 kV/mm_/G′_ο_ is the storage modulus sensitivity.

## 3. Experimental Section 

### 3.1. Materials

**Scheme 1:**
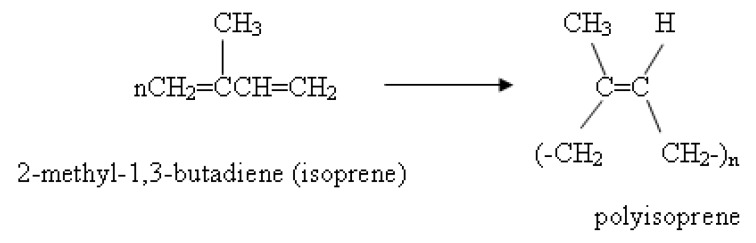
Polyisoprene structure.

Aluminum oxide (Al_2_O_3_) was purchased from Sigma-Aldrich Chemical Co., Ltd. (USA). The specific gravity of the alumina powder was 3.90 g/cm^3^. Hexane (HPLC grade) was obtained from Lab-Scan Co., Ltd. The starting natural rubber (STR XL or Standard Thai Rubber XL, STR XL, XL) was supplied by Venus Technology Co., Ltd. (VTEC, Thailand). STR XL is a dried rubber type in an extra light color slab and with a low dirt content of less than 0.04%. The specific gravity, impurity content, Mooney viscosity, initial plasticity, and plasticity retention of natural rubber (STR XL) are 0.92 g/cm^3^, 0.02% by weight, 62.8, 32.0, and 81.3, respectively. This natural rubber (STR XL) exhibits a high compounded gum tensile strength, good hot tensile properties, and possesses low level non-polymer constituents [5,11,26]. The chemical structure is shown in Scheme 1.

### 3.2. Instruments

Fourier transform infrared spectra (FTIR) were recorded on a Perkin Elmer, model Spectrum One spectrometer with a spectral resolution of 4 cm-1. The samples were mixed with the single-crystal potassium bromide (KBr).. X-ray diffraction (XRD) data were taken and analyzed using a Bruker, D8 Discover analyzer with a VANTEC-1 detector and a double-crystal wide-angle goniometer. Scans were obtained from 10° to 80° 2θ at a scan speed of 5° 2θ/min in 0.05° or 0.03° 2θ increments using CuKα radiation (λ = 0.154 nm). Peak positions were compared with standard JCPDS files to identify the crystalline phases. SEM micrographs were obtained using a scanning electron microscope (SEM, JEOL-5200) equipped with EDS for X-ray microanalysis. The samples of natural rubber XL, XL/Al2O3, and alumina powder (Al2O3) were mounted on a stub using carbon paste and were sputter-coated to ~0.1 µm of gold to improve conductivity. An acceleration voltage of 20 kV with magnifications of 1000 and 3200 times were used. The electrical properties were measured and obtained using an impedance analyzer (HP, model 16451B) equipped with an LCR meter (HP, model 4284A). The samples were prepared according to the ASTM B263-94 standard for the electrical property measurement. Composite samples were prepared as thin discs having a diameter of 38 mm and a thickness of 0.50 mm. In our experiment, the electrical properties were measured at frequencies from 500 to 106 Hz with an AC current of 2 A for samples at various volume fractions: Φ = $\frac{v_{disperse}}{v_{total}}$, where *Φ* is the volume fraction of the dispersed phase or alumina (Al_2_O_3_), *V_diperse_* is the volume of the dispersed phase (Al_2_O_3_), and *V_total_* is the total volume of the dispersed phase (Al_2_O_3_) and the matrix (STR XL) phase. 

The measured electrical resistivity of the composite materials was converted to electrical conductivity as follows [27]:

$$\rho_{eff}=\frac{RA}{L}=\frac{1}{\sigma }$$

where *ρ_eff_* is the effective resistivity (Ω.m), *R* is the resistance (Ω), *A* is the cross-section discs (m^2^), and *L* is the thickness (m).

A controlled-strain rheometer (Rheometric Scientific Inc., ARES) was used to measure the dynamic rheological properties of the composites under controlled strain with a copper parallel plate geometry (25 mm in diameter). The typical sample thickness of the parallel plate gap was 1.0 ± 0.1 mm. An electrical field for the ER measurement was applied using a high voltage power supply (Keithley, model 2410). Strain sweep tests were first carried out to determine the suitable strains to measure as functions of frequency at various DC electrical field strengths. The samples were pre-sheared at a low frequency (0.04 rad/s) with the electrical field on for 9 min in order to attain the equilibrium polarization. Each measurement was carried out at 27 °C and was repeated at least two or three times. Cumulative and fractional distributions were measured by using a particle size analyzer (Mastersizer S, model Polydisperse 2.19). The samples were dispersed in a water medium and vibrated in an ultrasonic cleaner for 20 min. 

Bulk densities of the alumina particles, the elastomer, and the composites were determined using a pynconometer by weighing the samples of known volumes with an accurate 4-digit balance. The composite densities were calculated from*ρ_C_* = *ρ_p_* Φ *+ ρ_m_* (1-Φ), where *ρ*_c,_ *ρ_p_* and *ρ_m_* are the densities (g/cm^3^) of the composites, the alumina, and the elastomer, respectively. Chemical compositions were obtained using an Oxford, model ED2000 X-ray fluorescence spectrometer (XRF), with a tube current of 1000 µA and an acquisition lifetime of 30 s. 

### 3.3. Sample Preparation of ER Solid

ER suspensions, with alumina particulate volume fractions of 0.00000 (XL/Al_0), 0.00054 (XL/Al_1), 0.00248 (XL/Al_2), 0.00448 (XL/Al_3), 0.00613 (XL/Al_4), 0.00750 (XL/Al_5), 0.00873 (XL/Al_6), 0.00968 (XL/Al_7), were prepared. The non-polar molecules tend to be soluble in non-polar solvents such as hexane, carbon tetrachloride, diethyl ether, and benzene. The dried natural rubber (XL) samples were swollen in a 20% by volume hexane solution. The hexane was HPLC grade with a density of 0.655 g/cm^3^, a relative permittivity of 2.0, and it is colorless. The alumina particles were dispersed using a magnetic bar stirrer at room temperature for 24 h. Each suspension was then poured into a Petri dish glass and allowed to dry at room temperature overnight. A thin disc with the thickness less than 0.5 mm and of light yellow in color was obtained. 

## 4. Conclusions 

The experimental data show that the XL/Al_7 composite with an Al_2_O_3_ particle volume fraction of 0.00968 has the highest electrical and electrorheological responses. The electrical properties, namely dielectric constant and electrical conductivity, of XL/Al_7 are 2.123 and 2.683 × 10^-8^ (Ω.m)-1, respectively. With and without an electrical field, the dynamic moduli G′ο, G′2kV/mm, G″ο, G″2kV/mm, and ∆G′/G′ο of the XL/Al_7 composite are 20011 Pa, 24388 Pa, 2827 Pa, 3639 Pa, and 21.88%, respectively. Our results suggest that small amounts of alumina (Al_2_O_3_) particles can be used as a filler to absorb loss and store additional elastic energy within the natural rubber matrix under electrical field. The XL/Al2O3 composites studied are soft and flexible materials and potential candidates for biomimetric actuators and/or artificial muscles.
